# Maternal hepatitis B e antigen can be an indicator for antiviral prophylaxis of perinatal transmission of hepatitis B virus

**DOI:** 10.1080/22221751.2021.1899055

**Published:** 2021-03-30

**Authors:** Ying Lu, Yarong Song, Xiangjun Zhai, Fengcai Zhu, Jianxun Liu, Zhanjun Chang, Yi Li, Yiwei Xiao, Lili Li, Minmin Liu, Jia Liu, Zhongping Duan, Huaibin Zou, Hui Zhuang, Jie Wang, Jie Li

**Affiliations:** a Department of Microbiology & Infectious Disease Center, School of Basic Medical Sciences, Peking University Health Science Center, Beijing, People's Republic of China; b Jiangsu Provincial Center for Disease Control and Prevention, Nanjing, People's Republic of China; c Zhengzhou Municipal Center for Disease Control and Prevention, Zhengzhou, People's Republic of China; d Beijing Youan Hospital, Capital Medical University, Beijing, People's Republic of China

**Keywords:** Hepatitis B virus, Mother-to-child transmission, Hepatitis B e antigen, Antiviral prophylaxis

## Abstract

As a high-risk factor of perinatal HBV transmission, the potential role of maternal hepatitis B e antigen (HBeAg) to guide antiviral prophylaxis has not yet been fully reported. This large prospective cohort study enrolled 1177 hepatitis B surface antigen (HBsAg)-positive pregnant women without antiviral treatment and their newborns. HBeAg, HBsAg, and viral load in maternal serum collected before delivery were measured. All the newborns were given standard passive–active immunoprophylaxis within 12 h after birth, and post-vaccination serologic testing was performed at 7 (±7d) months of age. The results revealed that 20 of the 1177 infants (1.70%) were immunoprophylaxis failure, and all their mothers were HBeAg positive. Maternal quantitative HBeAg was positively correlated with viral load (*r* = 0.83; *P* < .0001) and quantitative HBsAg (*r* = 0.68; *P* < .0001). The area under the receiver operating characteristic curve (AUC) for predicting immunoprophylaxis failure by maternal HBeAg was comparable to that by maternal viral load (0.871 vs 0.893; *P* = .441) and HBsAg (0.871 vs 0.871; *P* = .965). The optimal cutoff value of maternal quantitative HBeAg to predict perinatal infection was 2.21 log_10_ PEI U/mL, and the sensitivity and specificity was 100.0% and 74.5%, respectively. According to maternal viral load >2 × 10^5^ IU/mL, the sensitivity and specificity of maternal qualitative HBeAg to identify the risk of HBV MTCT for pregnant women and determine the necessity for antiviral prophylaxis was 95.5% and 92.6%, respectively. This study showed that maternal HBeAg can be a surrogate marker of HBV DNA for monitoring and evaluating whether antiviral prophylaxis is necessary for preventing perinatal HBV transmission.

## Introduction

Hepatitis B is a major global health problem and a leading cause of death. In 2015, an estimated 257 million people were living with chronic hepatitis B virus (HBV) infection worldwide, resulting in 0.8 million deaths attributed to cirrhosis and liver cancer [1]. In 2016, the World Health Organization (WHO) established an ambitious target to eliminate hepatitis B as a public health threat by 2030, aiming for a 95% decline in new cases of chronic infection and a 65% reduction in mortality [2]. Because of the effectiveness of vaccination in preventing HBV infection in later life, an increasing proportion of new infections is arising through mother-to-child transmission (MTCT), which is projected to rise from 16% in 1990 to 50% in 2030 [3]. Thus, prevention of HBV MTCT is a key priority in the combat against hepatitis B. Accordingly, the WHO also set a global target for coverage of interventions to prevent HBV MTCT from 38% in 2015 to 90% in 2030 [2].

In addition to birth dose vaccine, another approach like antiviral prophylaxis among pregnant women positive for hepatitis B surface antigen (HBsAg) should further reduce the risk of transmission [4,5]. Existing clinical practice guidelines have recommended antiviral prophylaxis for pregnant women at high risk of transmitting HBV to their infants, however, the standard screening protocol to identify HBsAg-positive pregnant women eligible for antiviral prophylaxis has not been established [6]. Various maternal HBV DNA thresholds (from 10^7^ to 10^8^ copies/mL) have been reported to be associated with perinatal transmission of HBV [7–11], but there is still no consensus on the optimal maternal viral threshold to initiate antiviral therapy solely to prevent HBV MTCT. Maternal quantitative HBsAg has also been found useful as a surrogate marker of HBV viral load and able to predict perinatal transmission of HBV [12–14]. Current guidelines from American Association for the Study of Liver Diseases (AASLD) and European Association for the Study of the Liver (EASL) recommend antiviral therapy solely to prevent HBV MTCT for pregnant women with high viral loads of >5.3 log_10_ IU/mL (>2×10^5^ IU/mL) [15,16]. Maternal quantitative HBsAg (>4 log_10_ IU/mL) has also been suggested later by EASL as an indicator for antiviral prophylaxis of perinatal transmission [16]. Like quantitative HBsAg, quantitative hepatitis B e antigen (HBeAg) has also been proposed as a surrogate for HBV covalently closed circular DNA (cccDNA) [17]. However, as a high-risk factor of perinatal HBV transmission, the potential role of maternal quantitative HBeAg to guide antiviral prophylaxis has rarely been reported.

Regarding the use of maternal quantitative HBV DNA and HBsAg in prenatal screening, the major concern is the feasibility and affordability of such quantitative tools in low- and middle-income countries (LMICs) where there is a lack of laboratory capacity in community or outreach settings and where access to appropriate diagnostics represents a greater financial obstacle than drugs. To decentralize antenatal screening and scale-up antiviral treatment of pregnant women in these contexts, it is critical to develop protocols based on easy-to-perform and cost-effective diagnostics to identify pregnant women at risk of transmission, since the cost of antivirals should no longer be the main barrier [18]. Maternal seropositivity for HBeAg is associated with high HBV replication and with an increased risk of HBV MTCT, and conceivably maternal qualitative HBeAg, without the need for cost-prohibitive laboratory infrastructures and trained personnel compared with HBV DNA and quantitative serology, could possibly be an alternative indicator to initiate antivirals in pregnant women [19]. Notably, a recent meta-analysis showed that maternal HBeAg could predict immunoprophylaxis failure in infants with high sensitivity and identify mothers with serum HBV DNA above 5.3 log_10_ IU/mL accurately [20]. On the basis of results of the meta-analysis, WHO recommends that HBeAg testing can be used as an alternative to HBV DNA testing to determine eligibility for tenofovir prophylaxis to prevent mother-to-child transmission of HBV [21]. Therefore, maternal HBeAg is a promising candidate to assess eligibility for antiviral prophylaxis awaiting further validation.

The purpose of this study was to evaluate the performance of maternal quantitative or qualitative HBeAg as an indicator for antiviral prophylaxis of perinatal HBV transmission and to frame the prenatal screening strategy to identify pregnant women at risk of transmission in different settings.

## Material and methods

### Subjects

**Figure 1.**
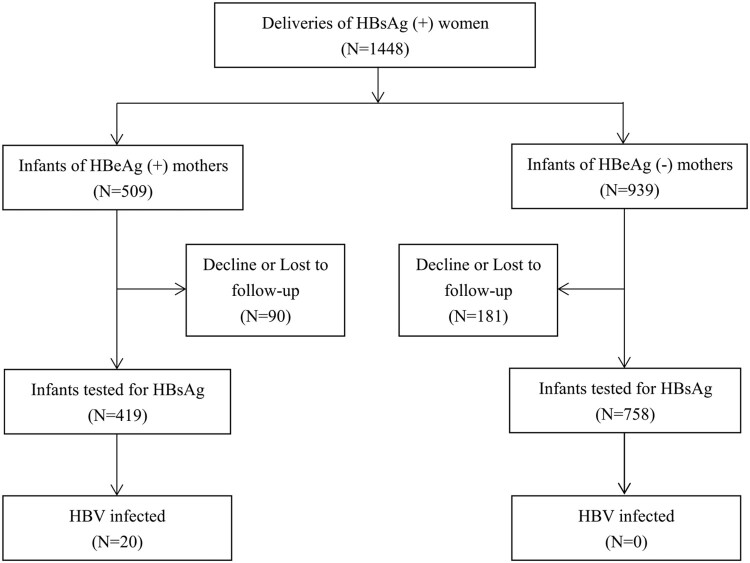
Enrollment and follow-up of participants.

This was a prospective cohort study consisting of pregnant women found to be chronically infected with HBV during antenatal visits at community maternal and child health centres in Jiangsu and Henan Provinces, China, and their babies. Pregnant women under antiviral therapy before or during pregnancy were excluded. Maternal HBeAg status was determined and maternal viral load, quantitative HBsAg and HBeAg were measured before labour. All infants received three doses of recombinant yeast-derived hepatitis B vaccine (HepB) (10 μg/0.5 mL or 20 μg/1.0 mL; Dalian Hissen Biopharm Inc., Dalian, China or Shenzhen Kangtai Biological Products Co., Ltd., Shenzhen, China) at birth (within 12 h), 1, and 6 months, combined with one dose of hepatitis B immunoglobulin (HBIG) (Hualan Biological Engineering Inc., Xinxiang, China) within 12 h of birth. The main findings, other eligibility criteria and detailed protocol of the study were reported elsewhere[8]. As shown in Figure 1, we enrolled 509 infants of HBeAg-positive mothers and 939 infants of HBeAg-negative mothers at birth. At 7 months, 419 (82.3%) infants of HBeAg-positive mothers and 758 (80.7%) infants of HBeAg-negative mothers were tested for transmission. Other details have been reported elsewhere [8]. The study was approved by the institutional review board of Peking University Health Science Center. Written informed consent was obtained from all participants.

### Laboratory evaluation

Maternal viral load was quantified by Abbott real-time HBV DNA assay (Abbott Molecular, IL, USA) using Abbott m2000 system (with the lower detection limit of 1.18 log_10_ IU/mL). Maternal quantitative HBsAg and semi-quantitative HBeAg were measured by Abbott Architect i2000 chemiluminescent microparticle immunoassay (CMIA) (Abbott Diagnostic, Chicago, IL, USA). Assay results for HBeAg were reported as the ratio of sample relative light unit (RLU) to cut-off RLU (S/CO). Samples with S/CO lower than 1.0 were considered negative for HBeAg. Maternal quantitative HBeAg was calculated by the formula $C=10^{\left[(\log_{10}S/CO\;-\;0.6977)/0.94\right]}$ PEI U/mL, a curve plotted for S/CO index using Paul Ehrlich Institute (PEI) standards, if maternal HBeAg S/CO was lower than 350. In other cases, samples were further diluted for retesting and recalculation.

### Polymerase chain reaction, sequencing and the phylogenetic analysis

HBV DNA extracted from 200 µL of serum samples using QIAamp DNA blood mini kit (Qiagen, Hilden, Germany) was used for the amplification of full-length HBV genome by PCR, as described by a previous study of our group, followed by direct sequencing [22]. HBV full-length genome was successfully amplified and sequenced in 15 mother-infant pairs (failed amplification and direct sequencing in the other 5 mother-infant pairs due to insufficient serum). Sequence alignment was completed and neighbour-joining phylogenetic tree was constructed by MEGAX software. The sequence homology search was done using the BLAST programme at NCBI. The full-length HBV genome was aligned with the reference sequence (genotype C, GenBank accession no. AB014378).

### Definition

Immunoprophylaxis failure was defined as positive HBsAg and HBV DNA for infants at 7 months old.

## Statistical analyses

Statistical analyses were done by SPSS statistical package (version 24.0). Categorical variables were expressed as a proportion (%, n/n) and examined by Chi-square or Fisher’s exact test. Continuous data were expressed as median (range) and compared by Mann–Whitney *U-*test. The correlation between maternal quantitative HBV DNA and quantitative serology was examined by Spearman correlation coefficient. Area under the receiver operating characteristic (ROC) curve (AUC) was used to evaluate the performance of various markers to predict infant infection and to identify mothers at risk of HBV MTCT and eligible for antiviral prophylaxis. The Youden’s index was calculated to determine the optimal cut-off. The ROC contrast test was used to compare ROC curves.

## Results

### Maternal characteristics

Table 1 shows maternal characteristics by HBeAg status. HBeAg-positive mothers were younger (24.0 vs 26.7 years; *P* < .0001) and had higher HBV DNA (8.12 vs 2.69 log_10_ IU/mL; *P* < .0001) and HBsAg levels (4.39 vs 3.34 log_10_ IU/mL; *P* < .0001) than HBeAg-negative mothers. The median HBeAg titre was 3.07 log_10_ PEI U/mL.

**Table 1.** Maternal characteristics by HBeAg status.

	Overall	HBeAg+	HBeAg–	*P^a^*
*N* (%)	1177	419 (35.6)	758 (64.4)	–
Age (yrs), median (range)	26.0 (15.1–43.0)	24.0 (16.5–43.0)	26.7 (15.1–43.0)	<.0001
HBV DNA (log_10_ IU/mL), median (range)	3.33 (1.18–9.22)	8.12 (1.26–9.22)	2.69 (1.18–8.68)	<.0001
HBsAg (log_10_ IU/mL), median (range)	3.64 (−1.30–5.03)	4.39 (1.29–5.03)	3.34 (−1.30–4.71)	<.0001
HBeAg (log_10_ PEI U/mL), median (range)	3.07 (−0.74–-4.06)	3.07 (−0.74–4.06)	–	–

^a^ *P* values represented the statistical differences between HBeAg-positive and HBeAg-negative mothers, which were calculated by Mann–Whitney *U* test.

The distributions of maternal quantitative HBsAg in all and by HBeAg status are depicted in Supplementary Figure 1. High HBsAg titres of ≥4 log_10_ IU/mL were found in 32.9% (387/1177) of all HBsAg-positive mothers. A significantly higher proportion of HBeAg-positive mothers had HBsAg titres ≥4 log_10_ IU/mL compared with HBeAg-negative mothers (71.4%, 299/419 vs 11.6%, 88/758; *P* < .0001). The distribution of maternal viral load in all and by HBeAg status has been reported in previous work of our team [8]. Among HBeAg-positive mothers, 76.1% (319/419) had HBeAg titres of ≥2 log_10_ PEI U/mL (Supplementary Figure 1). Maternal quantitative HBeAg had a positive correlation with both maternal viral load (*r* = 0.83, *P* < .0001) and quantitative HBsAg (*r* = 0.68, *P* < .0001) (Supplementary Figure 2).

### Perinatal transmission

Perinatal infection was confirmed in 20 infants of HBeAg-positive mothers (Figure 1). Homology analysis showed that full-length HBV strain from the mother was extremely close to that of her infant in all the 15 mother–infant pairs, with an average nucleotide homology of 99.5% (a homology of 100% in 7 mother-infant pairs, and 99% in the other 8 pairs) and an average genetic distance of 0.0002 (Supplementary Table 1). Neighbour-joining phylogenetic tree of HBV full-length genome was constructed for 15 mothers and their infants. All the mother-infant pairs were infected with genotype C, since all sequences clustered together with the reference sequence of genotype C HBV (GenBank accession No. AB014378). The results also showed that HBV sequences from each mother-infant pair clustered together, suggesting the existence of a close phylogenetic relationship between HBV sequences from the mother and her infant (Supplementary Figure 3).

All of the infected infants were born to mothers with HBV DNA levels >7 log_10_ IU/mL and HBsAg titres >4 log_10_ IU/mL, except one infected infant with a relatively lower maternal HBsAg titre of 3.92 log_10_ IU/mL. Perinatal transmission occurred in none of the infants born to HBeAg-negative mothers, even in those with high maternal HBV DNA levels of ≥7 log_10_ IU/mL (rate of infection: 0.0%, 0/5) and with high maternal HBsAg titres of ≥4 log_10_ IU/mL (rate of infection: 0.0%, 0/88). In HBeAg-positive mothers, as shown in Table 2, the mothers who transmitted the virus to their babies had higher HBV DNA levels (8.38 vs 8.12 log_10_ IU/mL; *P* = .004), higher HBeAg (3.12 vs 3.06 log_10_ PEI U/mL; *P* = .047) and HBsAg titres (4.50 vs 4.37 log_10_ IU/mL; *P* = .023) than the mothers who did not transmit the virus.

**Table 2.** Maternal characteristics of infants born to HBeAg-positive mothers by the outcome of immunoprophylaxis.

	Immunoprophylaxis success	Immunoprophylaxis failure	*P*
*N* (%)	392	20	
Age (yrs), median (range)	24.0 (16.5–43.0)	23.5 (18.0–32.0)	.095
HBsAg (log_10_ IU/mL), median (range)	4.37 (1.29–5.03)	4.50 (3.92–4.83)	.023
HBeAg (log_10_ PEI U/mL), median (range)	3.06 (−0.74–4.06)	3.12 (2.21–3.58)	.047
HBV DNA (log_10_ IU/mL), median (range)	8.12 (1.26–9.13)	8.38 (7.82–9.22)	.004
ALT (U/L), median (range)	18.00 (2.00–96.00)	16.00 (7.20–55.00)	.634

At maternal HBsAg titres of <3.50, 3.50–3.99, 4.00–4.49 and ≥4.50 log_10_ IU/mL, the rates of infection were 0.0% (0/511, 95% CI: 0.0–0.9), 0.4% (1/279, 95% CI: 0.0–2.2), 4.0% (9/223, 95% CI: 1.5–6.6) and 6.1% (10/164, 95% CI: 2.4–9.8), respectively. At maternal HBeAg titers of <2.00, 2.00–2.49, 2.50–2.99, 3.00–3.49 and ≥3.50 log_10_ IU/mL, the rates of infection were 0.0% (0/99, 95% CI: 0.0–4.5), 6.7% (1/15, 95% CI: 0.0–31.8), 4.4% (3/68, 95% CI: 1.0–12.7), 6.5% (13/199, 95% CI: 3.1–10.0) and 9.4% (3/32, 95% CI: 2.5–25.0), respectively. The rates of perinatal transmission at different maternal viral loads was reported previously[8].

Table 3 lists the risk factors for HBV MTCT. In univariate analysis, younger maternal age (OR for per 1-yr increase, 0.84; 95% CI: 0.74–0.95; *P* = .006), higher maternal quantitative HBsAg (OR for per log_10_ IU/mL increase, 18.51; 95% CI: 5.34–64.17; *P* < .001), higher semi-quantitative maternal HBeAg (OR for per log_10_ S/CO increase, 7.04; 95% CI: 1.26–39.40; *P* = .026), and higher maternal viral load (OR for per log_10_ IU/mL increase, 3.68; 95% CI: 1.53–8.89; *P* = .004) were associated with a higher risk of infection in infants. In multivariate analysis, maternal quantitative HBsAg, maternal semi-quantitative HBeAg and maternal viral load were analysed separately in model 1, model 2 and model 3 because multicollinearity would occur if highly correlated variables were used in the same model. After adjustment for maternal age, infants with higher maternal HBsAg (adjusted OR for per log_10_ IU/mL increase, 17.34; 95% CI: 4.75–63.26; *P* < .001), higher maternal HBeAg (adjusted OR for per S/CO increase, 6.78; 95% CI: 1.14–40.30; *P* = .035) or higher maternal viral load (adjusted OR for per log_10_ IU/mL increase, 3.78; 95% CI: 1.46–9.81; *P* = .006) had a significantly higher risk of infection.

**Table 3.** Univariate and multivariate logistic regression analyses of factors related to immunoprophylaxis failure.

Variable	Univariate		Multivariate					
			Model 1		Model 2		Model 3	
	OR (95% CI)	*P*	Adjusted OR (95% CI)	*P*	Adjusted OR (95% CI)	*P*	Adjusted OR (95% CI)	*P*
Maternal age (per 1-year increase)	0.84 (0.74–0.95)	.006	0.88 (0.78–1.00)	.054	0.90 (0.79–1.03)	.113	0.89 (0.78–1.02)	.081
Maternal HBsAg (per log_10_ IU/mL increase)	18.51 (5.34–64.17)	<.001	17.34 (4.75–63.26)	<.001	–	—	—	—
Maternal HBeAg (per log_10_ S/CO increase)	7.04 (1.26–39.40)	.026	—	—	6.78 (1.14–40.30)	.035	—	—
Maternal viral load^a^ (per log_10_ IU/mL increase)	3.68 (1.53–8.89)	.004	—	—	—	—	3.78 (1.46–9.81)	.006
Maternal ALT > ULN (40 U/L) vs. ≤ ULN	0.65 (0.08–4.95)	.673						
Maternal HBV genotype^b^ C vs. B	0.59 (0.19–1.79)	.349						
Male vs. female newborn	0.96 (0.40–2.32)	.924						
Birth weight (per 1-kg increase)	0.87 (0.30–2.51)	.799						
Cesarean vs. vaginal birth	0.61 (0.25–1.51)	.287						
Breast^c^ vs. formula feeding	0.96 (0.39–2.36)	.924						

^a^ This category excluded 96 mothers negative for serum HBV DNA.

^b^ This category excluded 319 mothers with insufficient sera or low viral loads and the genotype could not be determined. This category also excluded 18 mothers infected with mixed genotypes and 7 with genotype D.

^c^ The breastfeeding group consists of infants fed breast milk exclusively and those fed both breast milk and formula.

### Maternal quantitative/semi-quantitative HBeAg to predict infection

**Figure 2.**
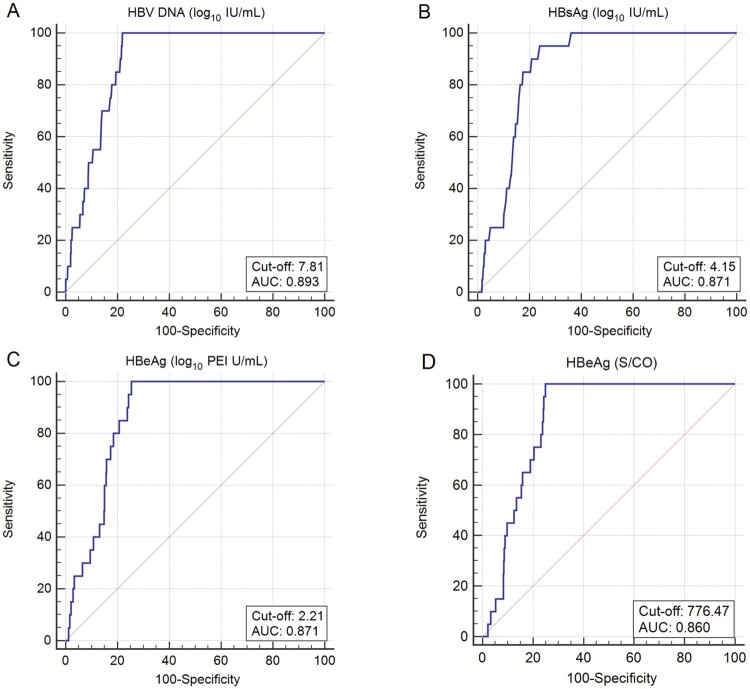
ROC curves for maternal viral load, quantitative HBsAg and quantitative/semi-quantitative HBeAg to predict infant infection. (A) ROC curves for maternal viral load to predict HBV infection in infants. (B) ROC curves for maternal quantitative HBsAg to predict HBV infection in infants. (C) ROC curves for maternal quantitative HBeAg to predict HBV infection in infants. (D) ROC curves for maternal semi-quantitative HBeAg to predict HBV infection in infants.

Figure 2(A, B and C) shows the performance of maternal viral load, quantitative HBsAg and HBeAg to predict infant infection. The AUC for maternal HBV DNA, quantitative HBsAg and HBeAg to predict infection was 0.893 (95% CI: 0.873–0.910; *P* < .0001), 0.871 (95% CI: 0.850–0.890; *P* < .0001), and 0.871 (95% CI: 0.850–0.890; *P* < .0001), respectively. The AUC for maternal quantitative HBeAg to predict infection was comparable to that for maternal viral load (*P* = .441) and quantitative HBsAg (*P* = .965) to predict infection. The optimal cut-off of maternal viral load to predict infection was 7.81 log_10_ IU/mL, with a sensitivity of 100.0% and a specificity of 77.9%. The optimal cut-off of maternal quantitative HBeAg to predict infection was 2.21 log_10_ PEI U/mL, with a sensitivity of 100.0% and a specificity of 74.5%. The optimal cut-off of maternal quantitative HBsAg to predict infection was 4.15 log_10_ IU/mL, with a sensitivity of 95.0% and a specificity of 76.0%.

We also examined the possibility of using maternal semi-quantitative HBeAg as an alternative to predict infant infection (Figure 2D). The AUC for maternal semi-quantitative HBeAg to predict infection was 0.860 (95% CI: 0.839–0.879; *P* < .0001) and the optimal cut-off of HBeAg S/CO was 776.47, with a sensitivity of 100.0% and a specificity of 75.1%. The AUC for maternal semi-quantitative HBeAg to predict infection was comparable to that for maternal quantitative HBeAg to predict infection (*P* = .484).

### Maternal quantitative/semi-quantitative HBeAg as an alternative of quantitative HBV DNA and HBsAg to assess eligibility for antiviral prophylaxis

**Figure 3.**
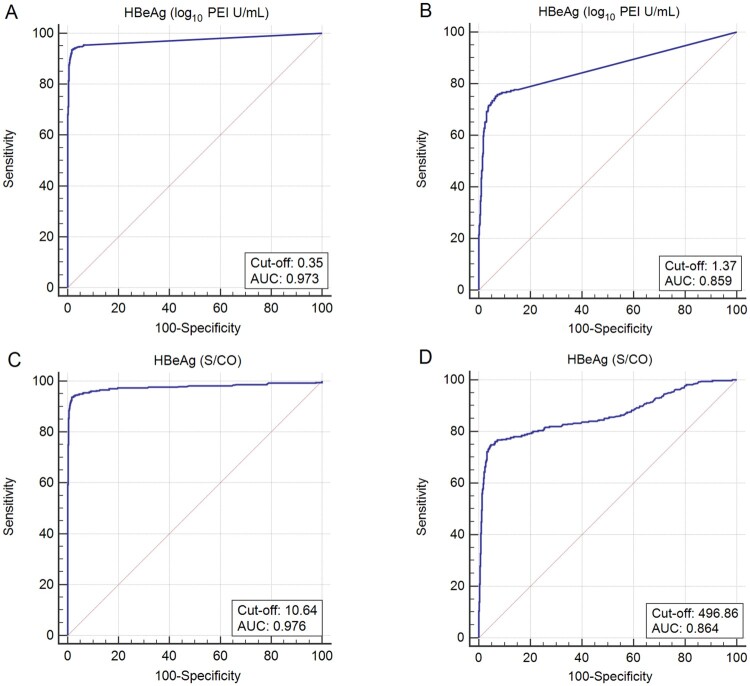
ROC curves for maternal quantitative/semi-quantitative HBeAg to predict maternal viral loads ≥5.3 log_10_ IU/mL and maternal HBsAg levels of ≥4 log_10_ IU/mL. ROC curves for maternal quantitative HBeAg to predict (A) maternal viral loads ≥5.3 log_10_ IU/mL and (B) maternal HBsAg levels of ≥4 log_10_ IU/mL. ROC curves for maternal semi-quantitative HBeAg to predict (C) maternal viral loads ≥5.3 log_10_ IU/mL and (D) maternal HBsAg levels of ≥4 log_10_ IU/mL.

Current guidelines from AASLD and EASL recommend antiviral therapy to prevent HBV MTCT for pregnant women with high viral loads (>5.3 log_10_ IU/mL) and high HBsAg titres (>4 log_10_ IU/mL)[15,16]. The performance of maternal quantitative HBeAg to predict maternal viral loads of ≥5.3 log_10_ IU/mL and HBsAg titres of ≥4 log_10_ IU/mL is shown in Figure 3(A,B), respectively. The predictive accuracy was high, with an AUC of 0.973 (95% CI: 0.962–0.982; *P* < .0001) and 0.859 (95% CI: 0.838–0.879; *P* < .0001) to predict maternal viral loads of ≥5.3 log_10_ IU/mL and HBsAg titres of ≥4 log_10_ IU/mL, respectively. The optimal cut-off of maternal HBeAg titre was 0.35 log_10_ PEI U/mL (sensitivity: 93.6%, specificity: 98.4%) and 1.37 log_10_ PEI U/mL (sensitivity: 75.3%, specificity: 93.3%) to predict maternal viral loads of ≥5.3 log_10_ IU/mL and HBsAg titres of ≥4 log_10_ IU/mL, respectively.

We also examined the possibility of using maternal semi-quantitative HBeAg to predict maternal viral loads of ≥5.3 log_10_ IU/mL and HBsAg titres of ≥4 log_10_ IU/mL. The AUC for maternal semi-quantitative HBeAg to predict maternal viral loads of ≥5.3 log_10_ IU/mL and HBsAg titres of ≥4 log_10_ IU/mL was 0.976 (95% CI: 0.966–0.984; *P* < .0001) and 0.864 (95%CI: 0.843–0.883; *P* < .0001), respectively (Figure 3C,D). The optimal cut-off of HBeAg S/CO was 10.64 to predict maternal viral loads of ≥5.3 log_10_ IU/mL, with a sensitivity of 93.6% and a specificity of 98.4%. The optimal cut-off of HBeAg S/CO was 496.86 to predict maternal HBsAg titres of ≥4 log_10_ IU/mL, with a sensitivity of 74.9% and a specificity of 95.2%. The performance of maternal semi-quantitative HBeAg to predict maternal viral loads of ≥5.3 log_10_ IU/mL (*P* = .455) and HBsAg titres of ≥4 log_10_ IU/mL (*P* = .645) was comparable to that of maternal quantitative HBeAg.

## Performance of maternal qualitative HBeAg to identify mothers eligible for antiviral prophylaxis according to different HBV DNA and HBsAg thresholds

The sensitivity and specificity of maternal qualitative HBeAg to detect an HBV DNA level of >2 × 10^5^ IU/mL, the viral threshold to initiate antiviral prophylaxis of HBV perinatal transmission suggested by AASLD and EASL guidelines, was 95.5% and 92.6%, respectively. The sensitivity and specificity according to various viral thresholds are reported in Table 4. The sensitivity and specificity of maternal qualitative HBeAg to detect an HBsAg titre of >4 log_10_ IU/mL, the indicator for perinatal antiviral therapy solely to prevent mother-to-child transmission of HBV in EASL guidelines, was 78.6% and 84.8%, respectively.

**Table 4.** The performance of maternal qualitative HBeAg to identify mothers eligible for antiviral prophylaxis according to different HBV DNA and HBsAg thresholds.

		HBeAg		Sensitivity (95% CI)	Specificity (95% CI)
Maternal HBV DNA threshold (log_10_ IU/mL)	HBV DNA*^a^*	Positive (*n* = 419)	Negative (*n* = 758)		
5	Positive	364	24	93.8% (91.4–96.2)	93.0% (91.3–94.8)
	Negative	55	734		
6	Positive	349	12	96.7% (94.8–98.5)	91.4% (89.5–93.3)
	Negative	70	746		
7	Positive	324	5	98.5% (96.4–99.5)	88.8% (86.7–90.9)
	Negative	95	753		
5.3 (2 × 10^5^ IU/mL)	Positive	360	17	95.5% (93.4–97.6)	92.6% (90.8–94.4)
	Negative	59	741		
6.3 (2 × 10^6^ IU/mL)	Positive	339	9	97.4% (95.8–99.1)	90.3% (88.3–92.4)
	Negative	59	741		
Maternal HBsAg threshold (log_10_ IU/mL)	HBsAg^*b*^				
4	Positive	298	81	78.6% (74.5–82.8)	84.8% (82.4–87.3)
	Negative	121	677		

^a^ “Positive” and “Negative” represented larger and less than maternal HBV DNA threshold, respectively.

^b^ “Positive” and “Negative” represented larger and less than maternal HBsAg threshold, respectively.

## Discussion

Identifying and treating highly viraemic pregnant women, whose infants are at risk of infection despite timely postnatal immunoprophylaxis, is a crucial step towards eliminating HBV MTCT. Our results show that maternal quantitative HBeAg of mothers who have never received antiviral therapy before pregnancy is positively correlated to maternal viral load and quantitative HBsAg, and predicts infant infection as well as maternal viral load or quantitative HBsAg does. To our knowledge, our study is the first to reveal the role of maternal quantitative HBeAg in predicting maternally transmitted infection and identifying mothers at risk of HBV MTCT. Moreover, our results indicate that maternal qualitative HBeAg has very high sensitivity and specificity to identify mothers with antiviral therapy naive at risk of HBV MTCT and eligible for antiviral prophylaxis according to different maternal viral load or HBsAg thresholds. These results are particularly significant for pregnant women in low-income countries, for qualitative testing of HBeAg is more affordable and easier to access comparing to quantitative HBV DNA.

**Figure 4.**
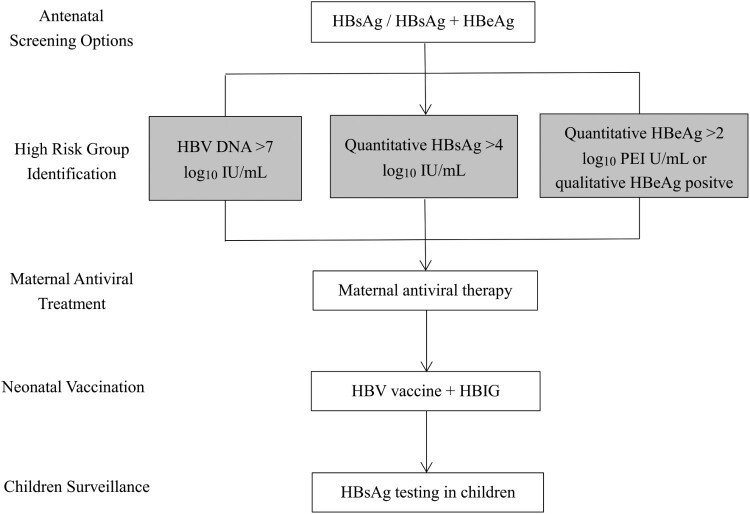
Proposed protocol for the management of pregnant women chronically infected with HBV and their babies.

Currently, existing clinical practice guidelines of academic societies and different countries have adopted maternal viral load or quantitative HBsAg as the indicator for antiviral prophylaxis of perinatal HBV transmission [15,16,23–25]. Commercial quantitative viral load or serology assays are becoming readily available, but the concern is how feasible it is, that is, the affordability and accessibility of diagnostics in most hospitals and primary health care facilities where the majority of antenatal care services are provided, especially in remote decentralized areas. Though quantitative serology assays are inexpensive (<10% of the cost of a quantitative HBV DNA assay) and use high-throughput platforms, they may still constitute a barrier to access to testing services in terms of financing, laboratory capacity and health workforce. Accordingly, our study provides evidence for risk stratification of HBV MTCT in different settings (Figure 4). In countries with abundant resources, following initial qualitative HBsAg testing, with or without sequential HBeAg testing, quantitative HBV DNA or quantitative serology can be used to determine eligibility for antiviral prophylaxis, with higher priority to qualitative HBeAg. As shown in our study, pregnant women with high viral load or quantitative serology should receive antiviral therapy to further reduce perinatal transmission, and the optimal thresholds for quantitative HBV DNA, HBsAg and HBeAg to predict perinatal infection are 7 log_10_ IU/mL, 4 log_10_ IU/mL and 2 log_10_ PEI U/mL, respectively. In countries with high HBV prevalence and poor access to quantitative HBV DNA or quantitative serology, the use of qualitative HBeAg testing, especially rapid diagnostic test (RDT) as a surrogate marker for quantitative HBV DNA or quantitative serology could be a rational trade-off for increased opportunities to identify pregnant women at high risk of transmission. Introduction and implementation of HBeAg RDT can be a cost-saving ($1.5 per test), point-of-care and timely (provides result in 15 min) approach to assess eligibility for antiviral prophylaxis in antenatal care [19]. It also has the potential to be integrated into the prenatal screening package together with human immunodeficiency virus and syphilis tests to be implemented up to primary health care facilities [19]. In this study, we find that maternal qualitative HBeAg may replace quantitative HBV DNA or serology to determine eligibility for antiviral prophylaxis, at least in China. Similar results have been reported in a recent study conducted in one hospital in Phnom Penh, Cambodia, which included 128 HBsAg-positive pregnant women. The sensitivity and specificity of SD BIOLINE HBeAg RDT in identifying highly viraemic samples was 76.5% and 96.8% for HBV DNA >5.3 log_10_ IU/mL, and 89.3% and 96.0% for HBV DNA >7.3 log_10_ IU/mL, respectively [19]. In Africa, another region with the heaviest burden of hepatitis B, a simple score based on HBeAg and ALT was developed to select patients for antiviral treatment and could potentially be adapted for identification of pregnant women at risk of transmission [18].

There are several other reasons for the consideration of using maternal qualitative HBeAg as an alternative indicator for antiviral prophylaxis. First, the presence of maternal HBeAg has been well recognized as an important risk factor for perinatal transmission. Maternal HBeAg has long been believed to induce immunotolerance in foetus and, clinically, is indicative of active viral replication, and is therefore correlated to an increased risk of HBV MTCT [26]. In the current study, maternal HBeAg status perfectly predicts the perinatal outcome. The cases with immunoprophylaxis failure were exclusively found with mothers positive for HBeAg. While HBeAg-negative mothers have a 0% risk of transmitting HBV to their babies with timely postnatal passive–active immunization. Actually, in the universal infant HBV immunoprophylaxis strategies of certain countries and areas, HBIG is optional for infants born to HBeAg-negative mothers, let alone the use of antivirals during pregnancy [27]. A very small proportion of HBeAg-negative pregnant women may have high viral loads above 5.3 log_10_ IU/mL (2.2%, 17/758, in our cohort), however, the risk of transmission is near 0% (0.23% reported in Taiwan [27], 0% reported in the US [9], and 0% in our cohort).

Second, antivirals given to HBeAg-positive pregnant women, regardless of their viral loads or HBsAg levels, may enable a greater safety margin. Taking our cohort for example, if a maternal HBsAg level of 4 log_10_ IU/mL is used as the cut-off according to existing practice guideline, one infected infant with a relatively lower maternal HBsAg level of 3.92 log_10_ IU/mL would be missed in such a protocol. Although all mothers of the infected infants would be identified and treated in this study if we use maternal viral loads of >2 × 10^5^ IU/mL as the eligibility criteria for receiving antiviral therapy, perinatal infection at a lower maternal viral load could not be totally ruled out in some cases, especially for mothers or infants with other possible risk factors for transmission, such as maternal-fetal haemorrhage or surface mutants with altered antigenicity [12]. Because perinatal acquisition of HBV often leads to higher lifetime risk for hepatitis B-related complications, a test with an excellent sensitivity and an acceptable specificity is more preferable. Evidence has supported a favourable safety profile for antiviral use in both mothers and infants [6]. Although post-partum hepatic flares have been observed in several studies and may be associated with the use of antiviral therapy, they are often mild in severity and most spontaneously resolve [28,29]. Also, no serious concerns regarding safety have been raised on antiviral therapy in asymptomatic carriers and immune tolerant individuals [30]. In our study, mothers positive for HBeAg slightly outnumbered mothers with viral loads of >2 × 10^5^ IU/mL or HBsAg levels of >4 log_10_ IU/mL. But the situation might be reversed in the case of the associated costs in real practice, after comprehensively considering the apparently lower costs of HBeAg qualitative testing, especially RDT, the expenses of specialized equipment, assays and technicians needed for quantitative HBV DNA or serology, and wider affordability and accessibility of antivirals.

Third, quantitative HBsAg in itself may not be an accurate indicator in mothers negative for HBeAg. In our cohort, 81 HBeAg-negative mothers had high HBsAg levels of >4 log_10_ IU/mL, but none of them were found with high viral loads of >2 × 10^5^ IU/mL, which indicated that HBsAg production might be relatively preserved and independent of viral load in HBeAg-negative mothers. That probably explains why the sensitivity for qualitative HBeAg to predict quantitative HBsAg >4 log_10_ IU/mL was relatively low.

In conclusion, maternal HBeAg can be a surrogate indicator of viral load for antiviral prophylaxis of HBV MTCT. Since HBeAg is easier to obtain and cheaper than viral load, it is suitable for more areas and populations. More large-scale prospective studies may be required in the future to further validate and confirm our results.

## Declarations

**Ethics approval and consent to participate:** The cohort data involved in the study was approved by the Bioethics Committees of Peking University. We confirm that we have all the necessary consents from any individuals involved in the study.

**Availability of data and materials:** The datasets used during the current study are available from the corresponding author on reasonable request.

**Author contributions:** Ying Lu and Yarong Song are joint first authors. Jie Li obtained funding. Jie Li, Jie Wang and Hui Zhuang designed the study. Ying Lu, Yarong Song, Yi Li, Yiwei Xiao, Lili Li, Minmin Liu and Jia Liu performed the study. Xiangjun Zhai, Fengcai Zhu, Jianxun Liu, Zhanjun Chang, Zhongping Duan and Huaibin Zou collected the serum samples. Ying Lu and Yarong Song analysed data and drafted the manuscript. Jie Li, Jie Wang and Hui Zhuang contributed to the interpretation of the results and critical revision of the manuscript for important intellectual content and approved the final version of the manuscript. All authors have read and approved the final manuscript. Jie Li, Jie Wang and Hui Zhuang are the study guarantors.

## Supplementary Material

Supplementary_material_-_revised_version.docx

## Abbreviations

**Abbreviations:** AASLD: American Association for the Study of Liver Diseases; AUC: area under the receiver operating characteristic curve; cccDNA: covalently closed circular DNA; CI: confidence interval; CMIA: chemiluminescent microparticle immunoassay; EASL: European Association for the Study of the Liver; HBeAg: hepatitis B e antigen; HBIG: hepatitis B immunoglobulin; HBsAg: hepatitis B surface antigen; HBV: hepatitis B virus; LMIC: low- and middle-income country; MTCT: mother-to-child transmission; PEI: Paul Ehrlich Institute; RDT: rapid diagnostic test; RLU: relative light unit; ROC: receiver operating characteristic; S/CO: sample to cut-off; WHO: World Health Organization
