# Supplementary material for: Maternal hepatitis B e antigen can be an indicator for antiviral prophylaxis of perinatal transmission of hepatitis B virus
Source: Emerg Microbes Infect. 2021 Mar 30;10(1):555–64. doi: 10.1080/22221751.2021.1899055 (PMC8018376; doi:10.1080/22221751.2021.1899055)
Supplement: Supplementary_material_-_revised_version.docx [file TEMI_A_1899055_SM8611.docx]

**Maternal Hepatitis B e Antigen can be an Indicator for Antiviral Prophylaxis of Perinatal Transmission of Hepatitis B Virus**

**Authors:** Ying Lu^1#^, Yarong Song^1#^, Xiangjun Zhai^2^, Fengcai Zhu^2^, Jianxun Liu^3^, Zhanjun Chang^3^, Yi Li^1^, Yiwei Xiao^1^, Lili Li^1^, Minmin Liu^1^, Jia Liu^1^, Zhongping Duan^4^, Huaibin Zou^4^, Hui Zhuang^1*^, Jie Wang^1*^, Jie Li^1*^

**
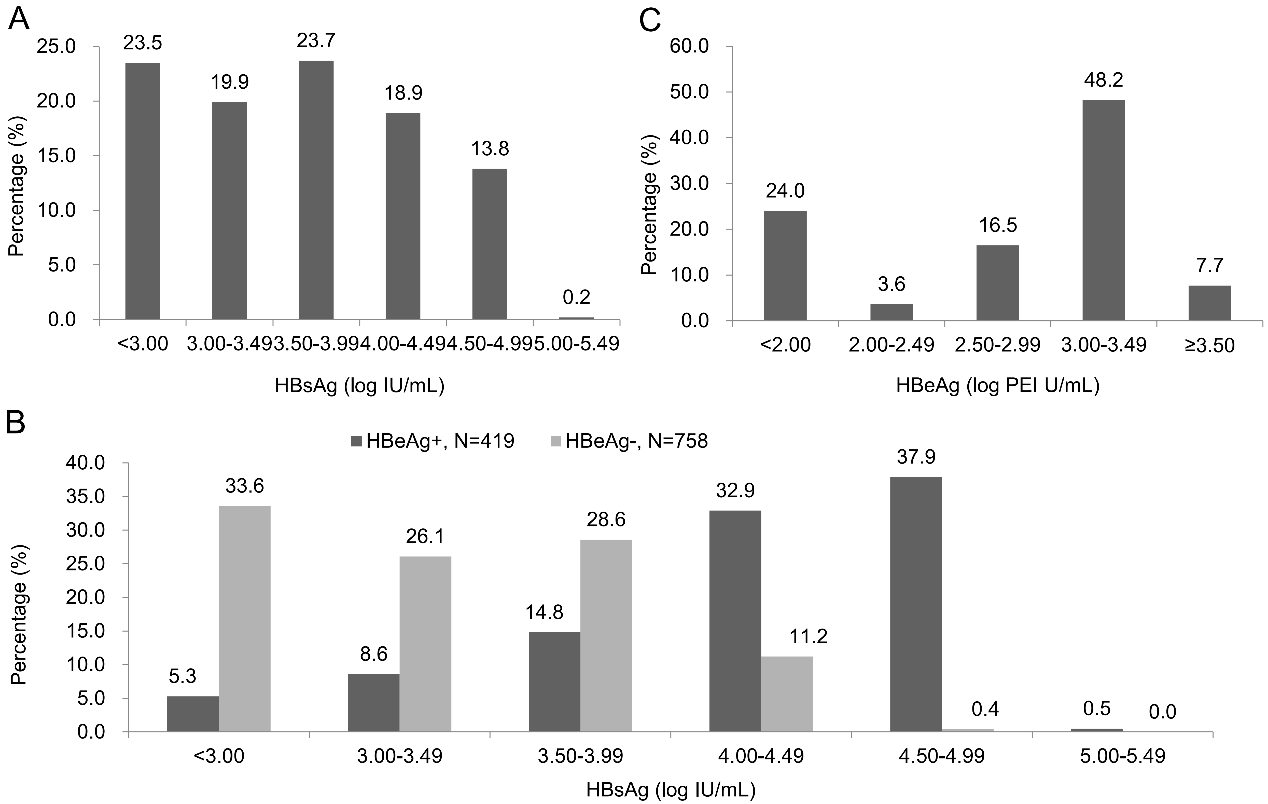
**

**Supplementary Figure 1.** Distribution of maternal quantitative HBsAg and HBeAg. (A) Distribution of serum HBsAg levels in all HBsAg-positive mothers. (B) Distribution of serum HBsAg levels by maternal HBeAg status. (C) Distribution of serum HBeAg levels in HBeAg-positive mothers.


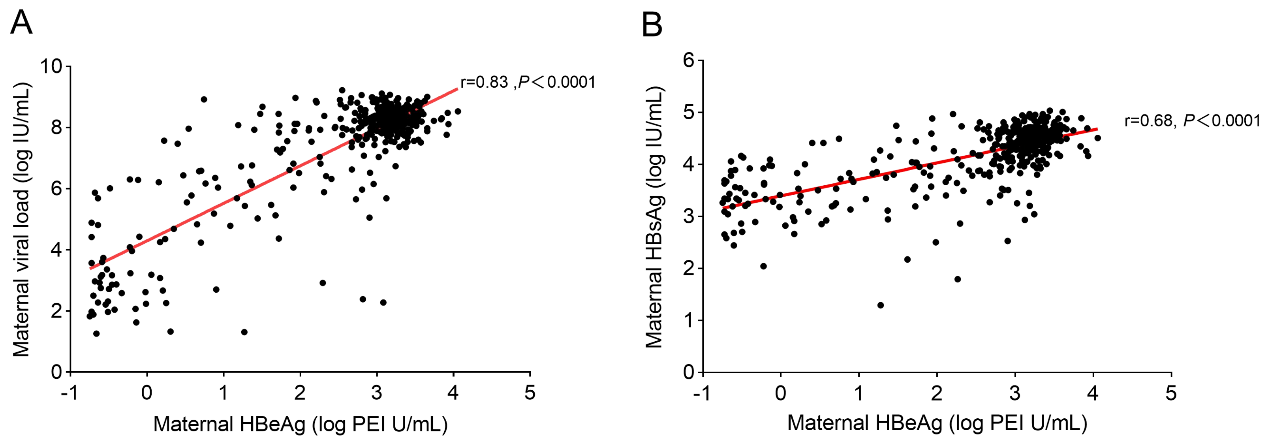


**Supplementary Figure 2.** Correlation between maternal viral load, quantitative HBsAg and HBeAg in HBeAg-positive mothers. (A) Correlation between maternal HBeAg and HBV DNA levels. (B) Correlation between maternal HBeAg and HBsAg levels.

**Supplementary Table 1.** Homology analysis of HBV full-length genome in 15 mother-infant pairs.

| Mother-infant pair | Genetic distance | Number of different sites | Homology |
| --- | --- | --- | --- |
| CZM076-CZC076 | 0.002 | 5 | 99% |
| CZM163-CZC163 | 0.000 | 0 | 100% |
| CZM229-CZC229 | 0.000 | 1 | 99% |
| CZM343-CZC343 | 0.000 | 1 | 99% |
| CZM452-CZC452 | 0.000 | 0 | 100% |
| TXM068-TXC068 | 0.001 | 3 | 99% |
| TXM184-TXC184 | 0.000 | 0 | 100% |
| TXM383-TXC383 | 0.000 | 0 | 100% |
| D043-DX043 | 0.000 | 1 | 99% |
| D106-DX106 | 0.000 | 1 | 99% |
| D110-DX110 | 0.000 | 0 | 100% |
| D117-DX117 | 0.000 | 1 | 99% |
| F062-FX062 | 0.000 | 0 | 100% |
| F080-FX080 | 0.000 | 1 | 99% |
| F136-FX136 | 0.000 | 0 | 100% |
| Mean ± SE | 0.0002 ± 0.0001 | 0.9333 ± 0.3581 | 99.5% ± 0.1% |


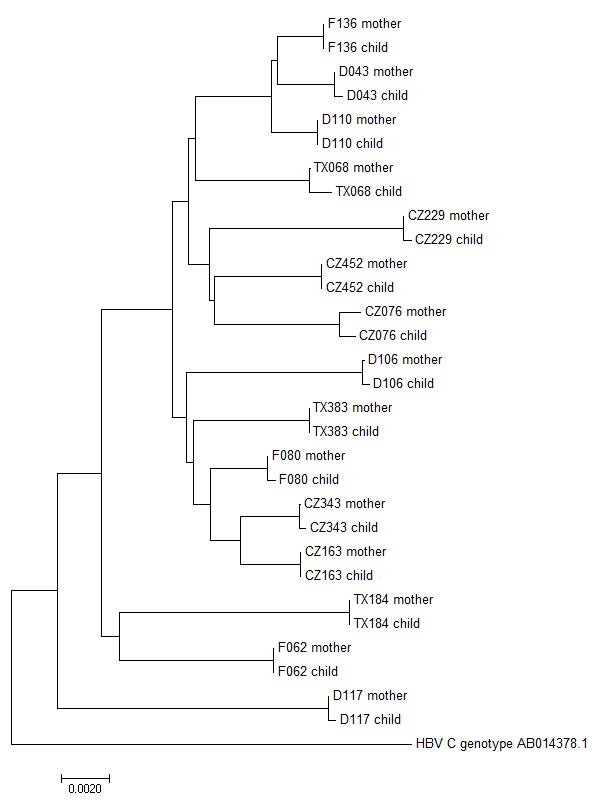


**Supplementary Figure 3.** The neighbor-joining phylogenetic tree of HBV full-length genome sequences in 15 mother-infant pairs. The full-length reference sequence of HBV genotype C was extracted from GenBank (accession No. AB014378).
